# Are People Living With Dementia Receiving High Intensity Statin Therapy After Stroke? A Population‐Based Cohort Study

**DOI:** 10.1002/hsr2.70165

**Published:** 2024-11-06

**Authors:** Leonie Picton, Johnson George, J. Simon Bell, Jenni Ilomäki

**Affiliations:** ^1^ Centre for Medicine Use and Safety, Faculty of Pharmacy and Pharmaceutical Sciences Monash University Parkville Victoria Australia; ^2^ School of Public Health, Faculty of Medicine, Nursing and Health Sciences Monash University Melbourne Victoria Australia

**Keywords:** dementia, linked data, statins, stroke

## Abstract

**Background and Aims:**

This Australian population‐based study investigated statin intensity after hospitalization for ischemic stroke in a matched cohort of people living with and without dementia.

**Methods:**

We identified all patients aged ≥ 30 years hospitalized in the state of Victoria, Australia, for ischemic stroke from July 1, 2013 to April 30, 2018 from the Victorian Admitted Episodes Data set. People with dementia were matched 1:4 for sex, 5‐year age group and index date ± 90 days with people without dementia. Records of statin dispensing within 60 days postdischarge were extracted from prescription claims data. The intensity of the first postdischarge statin dispensing was determined. Odds ratios for high versus low‐moderate intensity and no statin dispensing were estimated using multinomial logistic regression adjusted for factors including age, sex, and comorbidity.

**Results:**

The cohorts comprised 11,105 people (dementia: *N* = 2221; without dementia: *N* = 8884 and 52% were female. Compared to people without dementia, people with dementia had 35% (95% confidence interval [CI]: 24%–44%) lower odds of receiving a high intensity versus a low‐moderate intensity statin and 54% (95% CI: 48%–59%) lower odds of receiving a high intensity versus no statin. Compared to men, women with and without dementia had 16% (95% CI: 5%–25%) lower odds of receiving a high‐ versus low‐moderate intensity statin and 28% (95% CI: 19%–35%) lower odds of receiving a high intensity versus no statin.

**Conclusions:**

People living with dementia are less likely to receive high‐intensity statins post‐discharge compared to people without dementia. There is a gender gap in receipt of guideline‐recommended high‐intensity statin therapy for secondary prevention after ischemic stroke.

**Clinical Implications:**

Guidelines recommend all people with reasonable life expectancy receive a high‐intensity statin after stroke to reduce the risk of recurrent stroke and other adverse cardiovascular events. More research is needed to understand why people living with dementia might not receive guideline recommended care, and how statin use and statin intensity impact the health outcomes of people living with dementia and stroke.

## Abstract (highlights)

**What is Known:**

- High‐intensity statins provide the most benefit in reducing the risk of recurrent ischemic stroke or a cardiovascular event.
- People living with dementia do not always receive the same standard of care as those without dementia.

**What is New:**

- Compared to people without dementia, people living with dementia were 35% less likely to receive a high‐intensity statin versus a low‐moderate intensity statin and 54% less likely to receive a high‐intensity statin versus no statin.
- People living with dementia do not always receive the same standard of care as those without dementia.

## 1 Introduction

Dementia and stroke are leading causes of disease burden and death worldwide and in Australia [1, 2, 3]. Stroke mortality in Australia has fallen by 75% in recent decades, due to a number of factors including lower smoking rates, effective primary prevention measures, and advances in acute care of stroke [4]. People who have had a stroke have a 3%–5% annual risk of a future stroke [5]. Approximately 25% of strokes are recurrent strokes [6, 7].

The Australian and New Zealand Clinical Guidelines for Stroke Management recommend all patients with a reasonable life expectancy be prescribed a high‐intensity statin after stroke [8]. Statins have been shown to reduce the relative risk of recurrent ischemic stroke (IS) by 19% and cardiovascular events by 25%, with absolute risk differences of 1.6% and 5.4%, respectively [5]. High‐intensity statins provide the most benefit in reducing the risk of recurrent IS or a cardiovascular event [5, 9]. While there has been an evidence‐gap arising from exclusion of older people from clinical trials, more recent studies have found statins provide similar secondary prevention benefits in older and younger people [10, 11, 12, 13]. However, older people may be more susceptible to adverse drug events (ADEs), including myopathy, hepatotoxicity and new‐onset diabetes, particularly from high‐intensity statins [14]. In 2012, the United States Food and Drug Administration (FDA) published a Safety Announcement for all statins which included cognitive ADEs such as reversible memory loss and confusion, based on postmarketing case reports [15]. While the announcement included a statement from the FDA that the “cardiovascular benefits of statins outweigh the small increased risks,” fear of statin ADEs may have contributed to underuse despite the absolute risk of ADEs being small relative to the potential benefits [5, 16, 17]. There have also been conflicting reports on the potential of statins to reduce the risk of developing dementia, or slow cognitive decline in established dementia, which may also influence the decision on whether or not to use a statin [18, 19, 20, 21, 22].

The risk of recurrent stroke is highest soon after a stroke event, so preventive strategies should be implemented as soon as possible after IS [5]. Clinicians often seek to optimize secondary prevention regimens at the point of hospital discharge. Australian and Canadian studies have shown that prescribing preventive medications, including statins, at hospital discharge improves long‐term medication adherence [23, 24]. However, studies from Australia, the United Kingdom, and the United States of America suggest that people living with dementia do not always receive the same standard of care as those without dementia [25, 26, 27, 28].

Currently, little is known about how statins are prescribed after IS for people living with dementia. The objective of this population‐based study was to investigate statin intensity after hospitalization for IS in a matched cohort of people with and without dementia.

## 2 Methods

### 2.1 Study Design and Data Source

**Figure 1:**
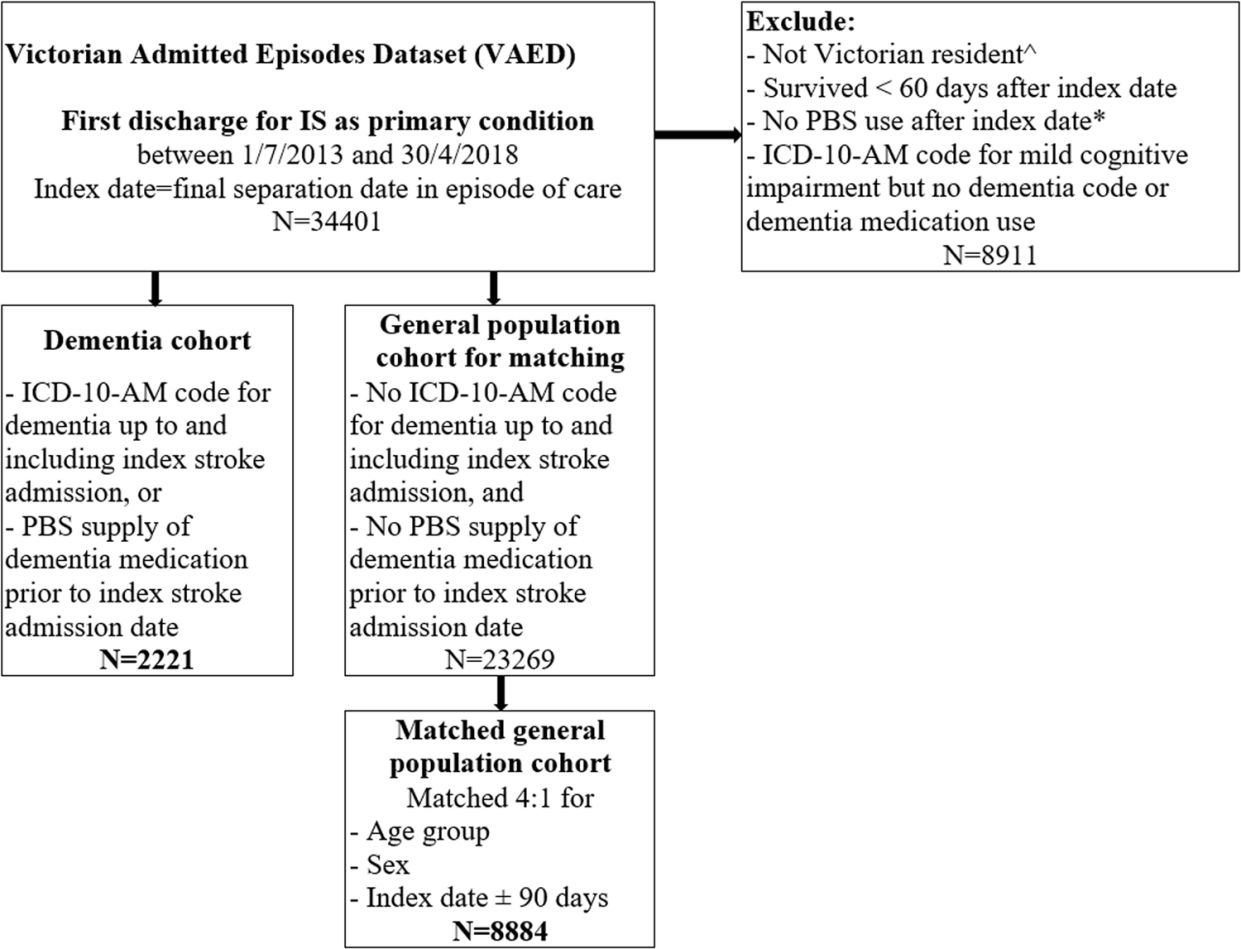
Cohort formation. ICD‐10‐AM, International Statistical Classification of Diseases and Related Health Problems, Tenth Revision, Australian Modification; IS, ischemic stroke; PBS, pharmaceutical benefits scheme. ^Victorian resident = code in VAED for Victorian residence during index stroke admission. * PBS use = at least 1 PBS supply for any item after index date.

This was a population‐based study investigating statin intensity after hospital discharge for IS, in a matched cohort of people living with and without dementia (Figure 1). We analyzed data from the Victorian Admitted Episodes Data set (VAED), National Death Index (NDI), and the Pharmaceutical Benefits Scheme (PBS) [29]. The VAED includes a minimum set of data reported by all public and private hospitals in the state of Victoria, Australia [30]. Variables in the VAED include admission and discharge dates, diagnoses, and patient characteristics (e.g., 5‐year age group, sex, marital status, preferred language, region of residence). The NDI includes all registered deaths across Australia. Key variables include date of death and primary cause of death. The PBS data set contains records of all prescriptions reimbursed under the national scheme for subsidized medications. Variables include PBS item code, date of supply, strength, and quantity dispensed. Data linkage and de‐identification were performed by the Australian Institute of Health and Welfare [31].

### 2.2 Cohort Definition

We identified all people aged ≥ 30 years discharged from a public or private hospital with IS as the primary condition from July 1, 2013 to April 30, 2018. IS was defined as the International Statistical Classification of Diseases and Related Health Problems, Tenth Revision, Australian Modification (ICD‐10‐AM) codes I63 or I64 [32]. The first admission for IS during the inclusion period was considered the index admission. The index date was the discharge date. If the episode of care included multiple admissions (e.g., transfer to a different ward), the index date was the date of the final discharge. People were included in our cohort if they were coded as Victorian residents at the time of the index admission, survived ≥ 60 days from the index date and had at least one PBS prescription dispensed for any item after the index discharge (Figure 1). We excluded people who survived ≤ 60 days postdischarge to exclude those potentially discharged for end‐of‐life care.

#### 2.2.1 People With Dementia

Dementia status was determined by presence of an ICD‐10‐AM code for dementia as a primary condition or comorbidity in the VAED records up to and including the index admission, or a PBS supply of an antidementia medication (donepezil, galantamine, rivastigmine, or memantine) or risperidone (when used to treat psychotic symptoms or aggression in people with Alzheimer's type of dementia) before the index admission date. The relevant ICD‐10‐AM, Anatomical Therapeutic Chemical (ATC), and PBS item codes are shown in Supporting Information S1: Table S1.

#### 2.2.2 People Without Dementia

People with IS and no record of dementia were matched for age group, sex, and index date (± 90 days) with a person with dementia. Each person with dementia was matched with a maximum of four people without dementia. People without dementia could be matched to > 1 person with dementia if they satisfied the matching criteria each time.

### 2.3 Statin Use and Intensity

Statins were classed as low, moderate, or high intensity according to the 2018 American College of Cardiology/American Heart Association Clinical Practice Guideline on the Management of Blood Cholesterol (Supporting Information S1: Table S2) [33]. Statin intensity was assessed before and after the index IS. The intensity of the first statin prescription dispensed on or up to 60 days after the index date was used to determine statin intensity after IS. The PBS item codes for statins are listed in Supporting Information S1: Table S3. The study period of 60 days was selected to provide a reasonable length of time to capture first postdischarge prescription dispensing. This was because some people have taken pre‐existing supplies from before or during their hospital admission before their first postdischarge statin dispensing. The PBS reimbursed statin prescriptions for a maximum 30‐day supply. The intensity of the last statin prescription dispensed in the 90 days before the index admission determined the intensity before IS. The 90‐day window before admission was chosen to accommodate lag‐time between dispensing and starting the new pack of medication, and varying levels of adherence.

### 2.4 Statistical Analyzes

Characteristics of people living with and without dementia were compared using descriptive statistics. If demographic data was missing in the index admission record, data recorded during other admissions were used to impute the data where possible. Charlson Comorbidity Index (CCI) scores were calculated using ICD‐10‐AM codes in the 365 days up to and including the index admission [34]. Statin use before and after IS was investigated by dementia status and age group using descriptive statistics. Odds ratios (OR) and 95% confidence intervals (95% CI) for using a high‐intensity statin versus no statin, a high‐intensity statin versus a low‐moderate intensity statin, or a low‐moderate intensity statin versus no statin were estimated using multinomial logistic regression models. Variables were chosen for consideration and initial analyses based on clinical relevance. Variables were included in the adjusted model based on clinical relevance and statistical significance in the unadjusted models. Variables included in the adjusted models were dementia status, sex, 5‐year age group, CCI score, previous stroke, marital status, area of residence (regional or metro), health insurance status (public or private), use of an interpreter, prior statin use and discharge destination (private residence, residential aged care facility [RACF], or other care setting). Variables considered but not included in the final models were admission source (private residence, RACF, or other care setting), preferred language (English or other), and hospital location, due to similarity with other included variables. Analyses were first performed in the overall cohort to estimate the role of dementia in predicting statin intensity. The analyses were then stratified by dementia status to investigate predictors of statin intensity in people with and without dementia. SAS 9.4 [TS1M6] (Cary, NC, USA) was used for all analyses.

### 2.5 Ethical Considerations

Ethics approval was granted by Monash University Human Research Ethics Committee (approval number 14339) and the Australian Institute of Health and Welfare Ethics Committee (approval number EO2018/4/468) for studies utilizing the linked data.

## 3 Results

### 3.1 Cohort Description

The matched cohort comprised 11,105 people (2221 with dementia; 8884 without dementia), aged over 30 years and 52% were female (Table 1). Overall, 45% of people were aged 85 years and over. However, 4% of people with dementia were under 65 years of age [35]. The comorbidity burden was similar across the cohorts; the median CCI score including dementia was 1‐unit higher for those with dementia compared to those without dementia (4 [interquartile range (IQR): 2–5] vs. 3 [IQR 1–4]) [34]. A higher proportion of people with dementia had had a previous stroke admission (12% vs. 7%). English was the preferred language for 84% of people with dementia and 88% of people without dementia, and 13% and 9%, respectively, required an interpreter. A slightly higher proportion of people with dementia resided in regional Victoria (75% vs. 71%), and more people with dementia were admitted from a RACF (6% vs. 2%). The median length of hospital stay for people with dementia was 15 days (IQR 5–32 days) and 13 days (IQR 4–31 days) for people without dementia. The proportion of people living in RACFs increased after IS for both cohorts with 32% of people with dementia and 10% of people without dementia being discharged to RACFs.

**Table 1:** Baseline information.

Patient characteristics	With dementia *N* (%)^b^	Without dementia *N* (%)^b^
*N*	2221	8884
Female	1150 (51.8)	4600 (51.8)
**Age**		
30–64	93 (4.2)	372 (4.2)
65–69	99 (4.5)	396 (4.5)
70–74	157 (7.1)	628 (7.1)
75–79	332 (15.0)	1328 (15.0)
80–84	541 (24.4)	2164 (24.4)
85+	999 (45.0)	3996 (45.0)
Charlson Comorbidity Index score median (IQR)	4 (2–5)	3 (1–4)
Previous stroke^c^	267 (12.0)	640 (7.2)
Current statin user^d^	875 (39.4)	4088 (46.0)
Most recent statin supply (if current user) (days before admission) median (IQR)	18 (9‐29)	18 (8‐29)
**Marital status (32 missing)**		
Married/de Facto	1066 (48.2)	4473 (50.5)
Single^a^	1147 (51.8)	4387 (49.5)
Interpreter required (22 missing)	284 (12.8)	780 (8.8)
Preferred Language English (252 missing)	1841 (83.5)	7604 (87.9)
**Location of residence**		
Metro	1659 (74.7)	6328 (71.2)
Regional	562 (25.3)	2556 (28.8)
**Hospital location**		
Metro	1697 (76.4)	6583 (74.1)
Regional	524 (23.6)	2301 (25.9)
Public patient	1548 (69.7)	5795 (65.2)
**Admitted from**		
Home^e^	1785 (80.4)	7670 (86.3)
Aged care	141 (6.4)	145 (1.6)
Other health care setting	295 (13.3)	1069 (12.0)
**Discharged to**		
Home^e^	1317 (59.3)	7346 (82.7)
Aged care	717 (32.3)	888 (10.0)
Other health care setting	187 (8.4)	650 (7.3)
Length of stay median (IQR)	15 (5–32)	13 (4–31)

Abbreviations: IQR, interquartile range; IS, ischemic stroke; Metro, metropolitan.

^a^ Single = never married, divorced, separated, or widowed.

^b^ *N* (%) unless otherwise stated.

^c^ Previous stroke = record of IS admission in Victorian Admitted Episodes Data set.

^d^ Statin supply within 90 days of admission.

^e^ Home = private residence.

### 3.2 Statin Intensity Before and After IS by Dementia Status

Before the index admission, 39% of people with dementia and 46% of people without dementia were dispensed statins (Supporting Information S1: Table S4). Postdischarge, 61% of people with dementia and 78% of people without dementia were dispensed statins. High‐intensity statin dispensing before IS was similar in people with and without dementia (16% and 19%, respectively), however, after IS, 41% of people with dementia and 59% of people without dementia were dispensed a high‐intensity statin. Use of low‐moderate intensity statins decreased slightly in both cohorts after IS (by 3% and 7%, respectively), to 20% of people with and without dementia. Most people with and without dementia who were using a high‐intensity statin before IS continued with a high‐intensity statin postdischarge (83% and 90%, respectively) (Supporting Information S1: Table S5). Of those who were using no statin before IS, 36% of people with dementia and 56% of people without dementia used a high‐intensity statin postdischarge, while 56% and 33%, respectively, continued to use no statin postdischarge. Among those who used no statin after IS, 1.4% and 2.7% of people with and without dementia, respectively, were dispensed ezetimibe (Supporting Information S1: Table S6).

**Figure 2:**
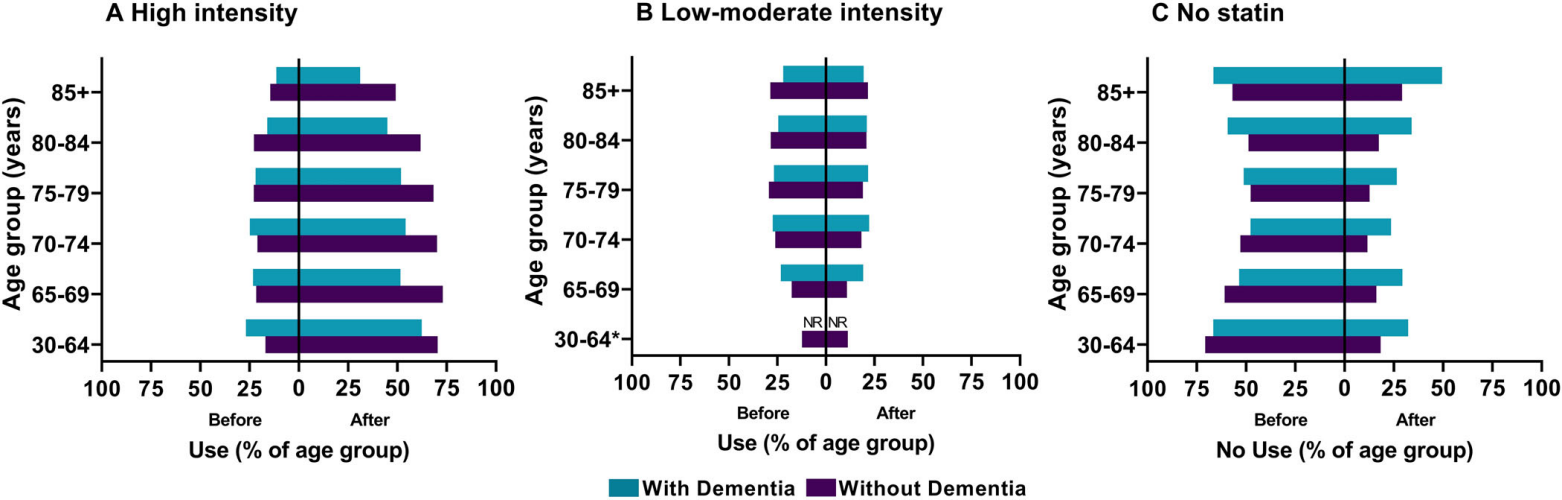
Statin use before and after ischemic stroke, by age group and dementia status. *NR = Not reported for dementia cohort due to low patient numbers in group.

In every age group, use of high‐intensity statins after IS was lower in people with dementia compared to those without dementia (Figure 2; Supporting Information S1: Table S4). The difference in high‐intensity statin use between those with and without dementia was smallest, eight percentage points (pp), in those under 65 years of age, and ranged from 16 to 21 pp in the other age groups. High‐intensity statin use decreased as age increased, particularly in those with dementia, with 45% of 80–84‐year‐olds and 31% of those over 85 years using a high‐intensity statin, compared to 62% and 49%, respectively, of corresponding age groups without dementia.

### 3.3 Statin Intensity After IS by Discharge Destination

Among people discharged to a RACF, 35% of people living with dementia were dispensed a high‐intensity statin compared to 43% of those without dementia. More people in RACFs with dementia were not using any statin compared to those without dementia (50% vs. 36%, respectively) (Supporting Information S1: Table S7). Similarly, among people discharged to a private residence, 45% of people living with dementia were dispensed a high‐intensity statin compared to 61% of those without dementia.

### 3.4 Dementia Status and Intensity of Statin Use After IS

People living with dementia were significantly less likely to receive any statin after IS compared to people without dementia (Table 2). Compared to people without dementia, people with dementia were 54% less likely to receive a high‐intensity statin than no statin (adjusted OR [aOR]: 0.46 [95% CI: 0.41–0.52]), 35% less likely to receive a high‐intensity statin than a low‐moderate intensity statin (aOR 0.65 [95% CI: 0.56–0.75]) and 29% less likely to receive a low‐moderate intensity statin than no statin (aOR: 0.71 [95% CI: 0.60–0.83]). See Supporting Information S1: Tables S8–S10 for full results of the regression models for the whole cohort.

**Table 2:** Odds of receiving a statin after ischemic stroke for people living with dementia compared to the matched people living without dementia.

Statin use after ischemic stroke	Odds ratio (95% CI)	
	Unadjusted	Adjusted^a^
High intensity versus no statin	0.39 (0.35–0.44)	0.46 (0.41–0.52)
High versus low‐moderate intensity statin	0.71 (0.63–0.81)	0.65 (0.56–0.75)
Low‐moderate intensity versus no statin	0.55 (0.48–0.63)	0.71 (0.60–0.83)

Abbreviation: CI, confidence interval.

^a^ Adjusted for age group, sex, Charlson comorbidity index score, prior statin use, previous stroke, marital status, area and type of residence, public/private patient status, and interpreter use.

### 3.5 Predictors of Statin Use in People With Dementia After IS

**Figure 3:**
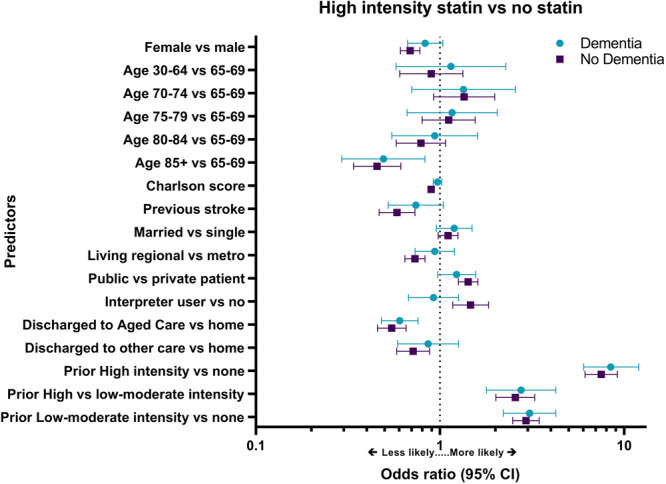
Predictors of using a high‐intensity statin versus no statin after ischemic stroke by dementia status. Charlson score = Charlson Comorbidity Index score; CI = confidence interval; Home = private residence; metro = metropolitan; see Supporting Information S1: Table S11 for odds ratios and 95% CIs.

In people with dementia, the strongest predictor of statin use after IS was prior statin use (Supporting Information S1: Table S11). Compared to people who used no statin before the index stroke, people who used a high‐intensity statin before IS were 8.4 times more likely to use a high‐intensity statin after IS than no statin (Figure 3) and 4.9 times more likely to use a high‐intensity statin after IS than a low‐moderate intensity statin (Supporting Information S1: Figure S1). People who were discharged to a RACF had significantly reduced odds of receiving any statin (high intensity vs. none: aOR 0.60 [95% CI 0.48–0.76]; low‐moderate intensity vs. none: aOR 0.55 [95% CI: 0.40–0.77]), but discharge to an RACF did not predict statin intensity among statin users (high vs. low‐mod: aOR: 1.09 [95% CI: 0.79–1.50]) (Figure 3, Supporting Information S1: Figures S1 and S2). Advanced age reduced the odds of being dispensed a high‐intensity statin by 51% in those over 85 years compared to those 65–69 years of age (aOR: 0.49 [95% CI: 0.29–0.82]). Women were 30% less likely than men to use a high‐intensity statin versus a low‐moderate intensity statin (aOR 0.70 [95% CI: 0.53–0.94]), however, sex was not a predictor of using any statin versus no statin. People who had had a previous stroke were less likely to use a high‐intensity statin than a low‐moderate intensity statin (aOR: 0.55 [95% CI: 0.36–0.83]). CCI score, marital status and use of an interpreter were not predictors of statin intensity.

### 3.6 Predictors of Statin Use in People Without Dementia After IS

Factors which may influence statin use after IS were similar in people without to those with dementia (Figure 3, Supporting Information S1: Figures S1 and S2). In people without dementia, prior statin use was also the strongest predictor of statin use after IS (Supporting Information S1: Table S11). Women were less likely to receive any statin than men (high intensity vs. none: aOR: 0.69 [95% CI 0.61–0.78]; low‐moderate intensity vs. none: aOR: 0.79 [95% CI: 0.67–0.92)], and of those who did receive a statin, women were less likely than men to use a high‐intensity statin versus a low‐moderate intensity statin (aOR: 0.87 [95% CI: 0.77–1.00]). Each one unit increase in CCI scores reduced the odds of statin use by 5%–11%. Publicly insured patients, and patients who used an interpreter were more likely to use a high‐intensity statin than a low‐moderate intensity statin (aOR: 1.45 [95% CI: 1.27–1.65] and aOR: 1.34 [95% CI 1.07–1.68], respectively) or no statin (aOR: 1.42 [95% CI: 1.26–1.61] and aOR: 1.47 [95% CI: 1.17–1.83], respectively). However, living in a regional area reduced the odds of using a high‐intensity statin (high intensity vs. none: aOR: 0.73 [95% CI: 0.64–0.83]; high vs. low‐moderate intensity: aOR: 0.77 [95% CI: 0.67–0.81]). People who had had a previous stroke were 42% less likely to use a high‐intensity statin versus no statin (aOR 0.58 [95% CI: 0.47–0.73]) and 40% less likely to use a high‐intensity statin than a low‐moderate intensity statin (aOR: 0.60 [95% CI: 0.47–0.77]).

## 4 Discussion

The main finding of this study was that people living with dementia were 54% less likely to use guideline recommended high‐intensity statins post‐discharge compared to people of the same age and sex without dementia. This suggests that many people living with dementia who survive a stroke do not receive effective secondary prevention medications. This is a concern because people with dementia have poorer outcomes after stroke [27, 36, 37]. Underutilization of statins and other secondary prevention measures may contribute to poorer poststroke health outcomes for some people living with dementia [25, 27].

While use of high‐intensity statins increased for both cohorts, statin intensity before IS was the strongest predictor of statin intensity after IS. Some patients may have appropriately been using a low‐moderate intensity statin or no statin before IS in accordance with guidelines for primary prevention, which take lipid levels into account [33]. However, guidelines for secondary prevention do not recommend statin intensity based on lipid profiles, instead recommending a high‐intensity statin for all, unless contraindicated or not tolerated [8, 33]. It is possible that some patients who used low‐moderate intensity or no statins after IS had previously not tolerated high‐intensity statins, or had favorable lipid profiles. The low uptake of the second‐line therapy ezetimibe, as monotherapy, suggests that contraindications to statins occur in a small proportion of patients.

The in‐hospital stay is an opportunity to counsel patients on their condition and ongoing care needs, set postdischarge goals of care, and initiate therapies for secondary prevention [23, 24]. Previous research suggests not all patients receive the optimal standard of care, particularly patients living with dementia, and this may result in patients leaving hospital without clear goals of care or preventive medications [27]. Reasons for the disparity in care are not clear, though clinical complexity, organizational challenges in delivering suitable care for dementia patients, attitudes of health workers towards people living with dementia and the lack of evidence for benefit in this population have been suggested as contributing factors [26, 27, 38]. It is also likely that individual patient experiences with statins, such as ADEs, and beliefs regarding the risks or benefits of medication may influence the decision to increase statin intensity, or continue as before with a low intensity or no statin, after the acute event [39]. Our data did not capture patient or physician beliefs and attitudes, however, a recent Australian study found that patients, carers, and clinicians had different preferences when considering whether to use antihypertensive medications for various indications including risk of stroke, in people living with dementia [40]. In that discrete choice experiment, people living with dementia showed a preference for decreasing pill burden, carers and clinicians showed a preference for reducing risk of future events while also considering the extent of cognitive decline, and clinicians preferred to reduce falls risk. Further qualitative research to investigate barriers and enablers of statin use after IS in people with dementia may assist in the development of educational packages and institutional policies to increase statin use in this population. Patients without dementia who used an interpreter had higher odds of using a statin, possibly due to the opportunity to clarify information and preferences for both clinicians and patients. More research is needed to understand how the way health information is delivered to patients influences their treatment decisions. Our results and another study from Australia show higher proportions of statin use after IS in people with and without dementia than in Sweden, however, the Swedish cohort was older (2007–2014) and statin use may have increased in Sweden since then [7, 27].

Patients discharged to RACFs may have different goals of care to those discharged to private residences. This may explain the lower prevalence of statin dispensing among these patients. Statins are often targeted for deprescribing in patients with limited life expectancy [41]. While a higher proportion of patients with dementia were discharged to RACFs in comparison to those without dementia (32% vs. 10%), among those from each cohort discharged to RACFs, fewer people living with dementia used a high‐intensity statin (35% vs. 43%, respectively), and more were not taking any statin (50% vs. 36%, respectively). Patients discharged to a private residence were likely to have better functioning, lower care needs, and a longer life expectancy than those discharged to RACFs. However, high‐intensity statin use in community‐dwelling people was still lower in people living with dementia (45% vs. 61%), with more people living with dementia receiving no statin (33% vs. 19%). Our data did not include information on functioning or mobility, so we were unable to investigate the impact of these factors on the decision to prescribe statins for secondary prevention.

Women with and without dementia were less likely to receive a high‐intensity statin than men. This was despite the Australian clinical guidelines recommending a high‐intensity statin for men and women. Our findings were consistent with United States data suggesting women have lower guideline‐recommended statin use for secondary prevention of cardiovascular events than men [39]. Living in a regional area reduced the odds of receiving a high‐intensity statin. This is consistent with other findings that people living in regional and remote areas have more limited access to healthcare and poorer outcomes than people living in metropolitan areas [6]. There is an urgent need to close the gap in healthcare between men and women, and by geographical location. Previous history of IS was also associated with lower odds of statin dispensing, possibly because many patients experience increased disability after recurrent stroke, and thus statins may not be prescribed due to changing goals of care. Given the large proportion of patients not receiving guideline recommended preventive medication, there remains scope for improving the rates of high‐intensity statin use after IS in patients with and without dementia.

## 5 Strengths and Limitations

The main strength of this study is the use of linked data, which gives investigators a richer picture of an individual's experience before, during, and after the episode of care, such as medication use and mortality information. The cohorts were extracted from mandated minimum data records of all hospitals serving a large state‐wide population base of over 6.5 million people, giving these results a high level of validity and generalizability [30]. Limitations of using administrative records for epidemiological research include a lack of clinical data, such as a previous adverse reaction to a statin and cholesterol levels, or life expectancy, which may impact prescribing. We were also unable to determine if statin use before the index stroke was for primary or secondary prevention. Even though limited data were available through ICD‐10‐AM codes from previous hospital admissions for some patients, comprehensive medical histories were not included in the data. It is likely that the non‐dementia cohort included some people with dementia because not everyone with dementia uses PBS‐subsidized medication, not all diagnosed dementia is coded during hospital admissions for other conditions, and there would be people in the community living with undiagnosed dementia [42]. We analyzed statin intensity based on PBS supply of and supply of a medication does not guarantee use. A change in health status postdischarge, such as developing post‐stroke dementia, may change goals of care, and statins may be reduced or ceased at that time. Our cohort was from 2013 to 2018 and high‐intensity statin use after IS may have increased since then.

## 6 Conclusion

People living with dementia are less likely to receive a high‐intensity statin after IS compared to their counterparts without dementia, even though current guidelines recommend all people with reasonable life expectancy receive a high‐intensity statin to reduce the risk of recurrent stroke and other adverse cardiovascular events. More research is needed to understand why people living with dementia might not receive guideline‐recommended care, and how statin use and statin intensity impact the health outcomes of people living with dementia and stroke.

## Author Contributions

**Leonie Picton:** conceptualization, data curation, formal analysis, investigation, methodology, visualization, writing–original draft, writing–review and editing. **Johnson George:** conceptualization, supervision, writing–review and editing. **J. Simon Bell:** conceptualization, funding acquisition, resources, writing–review and editing. **Jenni Ilomäki:** conceptualization, data curation, funding acquisition, methodology, project administration, resources, software, supervision, validation, writing–review and editing.

## Transparency Statement

The lead author Leonie Picton MClinEpid affirms that this manuscript is an honest, accurate, and transparent account of the study being reported; that no important aspects of the study have been omitted; and that any discrepancies from the study as planned (and, if relevant, registered) have been explained.

## Supporting information

Supporting information.

## Data Availability

The data underlying this article were provided via an application process by the Centre for Victorian Data Linkage and the Australian Institute of Health and Welfare and are not publicly available.
