# Supplementary material for: Are People Living With Dementia Receiving High Intensity Statin Therapy After Stroke? A Population‐Based Cohort Study
Source: Health Sci Rep. 2024 Nov 6;7(11):e70165. doi: 10.1002/hsr2.70165 (PMC11540808; doi:10.1002/hsr2.70165)
Supplement: Supplementary file 1 — Supporting information. [file HSR2-7-e70165-s001.docx]

# Supplementary Appendix

## Contents

Table S1. Diagnosis, ATC and PBS item codes

Table S2. Statin intensity

Table S3. Statin item codes

Table S4. Statin use and intensity before and after ischaemic stroke, by age group

Table S5. Consistency of statin intensity before and after IS

Table S6. Ezetimibe as monotherapy after IS

Table S7. Statin intensity after ischaemic stroke by discharge destination

Table S8. Odds of using a high intensity statin compared to no statin after ischaemic stroke

Table S9. Odds of using a high intensity statin compared to low-moderate intensity statin after ischaemic stroke

Table S10. Odds of using a low-moderate intensity compared to no statin after ischaemic stroke

Table S11. Predictors of statin intensity after ischaemic stroke by dementia status

Figure S1. Predictors of using a high versus a low-moderate intensity statin after ischaemic stroke by dementia status

Figure S2. Predictors of using a low-moderate intensity statin vs no statin after ischaemic stroke by dementia status

### Table S1. Diagnosis, ATC and PBS codes used to determine dementia status

| ICD-10-AM diagnosis codes for dementia | F00 F01 F02 F03 F051 G10 G20 G30 G31 A81 U791 |
| --- | --- |
| ICD-10-AM diagnosis codes for mild cognitive disorder | F067 |
| Dementia medication ATC codes | N06DA02 N06DA03 N06DA04 N06DX01 |
| PBS item codes for risperidone when used to treat symptoms of dementia | 01842Y 08787L 08788M 08789N 08790P 08791Q 09293D 11869Q 11872W 11873X 11874Y 11877D 11879F 11881H 11882J |

ATC = Anatomical Therapeutic Chemical classification; ICD-10-AM = International Statistical Classification of Diseases and Related Health Problems, Tenth Revision, Australian Modification; PBS = Pharmaceutical Benefits Scheme

### Table S2. Statin intensity

| **Statin** | **Low Intensity** | **Moderate Intensity** | **High Intensity** |
| --- | --- | --- | --- |
| Atorvastatin |  | 10mg, 20mg | 40mg, 80mg |
| Fluvastatin | 20mg, 40mg | 80mg |  |
| Pravastatin | 5mg, 10mg, 20mg | 40mg, 80mg |  |
| Rosuvastatin |  | 5mg, 10mg | 20mg, 40mg |
| Simvastatin | 5mg, 10mg | 20mg, 40mg | 80mg |

Statin intensity definitions from 2018 Cholesterol Clinical Practice Guidelines (Grundy et al^[[1]](#footnote-1)^) and adapted for the Australian context.

### Table S3. Statin codes

| Statin (ATC codes) | Strength | Intensity | PBS Item Codes |
| --- | --- | --- | --- |
| Atorvastatin  (C10AA05 C10BA05* C10BX03^) | 10mg | Moderate | 08213G 09230T 10392Y* 10002K* 09049G^ 09053L^ |
|  | 20mg | Moderate | 08214H 09231W 10393B* 02874G* 09050H^ 09054M^ |
|  | 40mg | High | 08215J 09232X 10377E* 02821L* 09051J^ 09055N^ |
|  | 80mg | High | 08521L 09233Y 10376D* 10006P* 09052K^ 09056P^ |
| Fluvastatin  (C10AA04) | 20mg | Low | 08023G 09234B |
|  | 40mg | Low | 08024H 09235C |
|  | 80mg | Moderate | 02863Q 09236D |
| Pravastatin  (C10AA03) | 5mg | Low | 02831B |
|  | 10mg | Low | 02833D 09237E |
|  | 20mg | Low | 02834E 09238F |
|  | 40mg | Moderate | 08197K 09239G |
|  | 80mg | Moderate | 08829Q 09240H |
| Rosuvastatin  (C10AA07 C10BA06*) | 5mg | Moderate | 02590H 02606E 03402C 09042X 10204C* |
|  | 10mg | Moderate | 02584B 02628H 03403D 09043Y 10208G* |
|  | 20mg | High | 02574L 02609H 03404E 09044B 10201X* |
|  | 40mg | High | 02594M 02636R 03405F 09045C 10207F* |
| Simvastatin  (C10AA01 C10BA02*) | 5mg | Low | 02013Y 09241J |
|  | 10mg | Low | 02011W 09242K 09483D* |
|  | 20mg | Moderate | 02012X 09243L 09484E* |
|  | 40mg | Moderate | 08173E 09244M 08881K* |
|  | 80mg | High | 08313M 09245N 08882L* |

ATC = Anatomical Therapeutic Chemical classification; PBS = Pharmaceutical Benefits Scheme; ~Statin intensity as defined in 2018 Cholesterol Clinical Practice Guidelines (Grundy et al) and adapted for the Australian context.

*Combination with ezetimibe

^Combination with amlodipine

### Table S4. Statin use and intensity before and after ischaemic stroke, by age group

| **Any statin** | **Dementia** | | | **No Dementia** | | |
| --- | --- | --- | --- | --- | --- | --- |
| Age group | Before (%) | After (%) | Change (%-point) | Before (%) | After (%) | Change  (%-point) |
| 30-64 | 33 | 68 | 35 | 29 | 82 | 53 |
| 65-69 | 46 | 71 | 25 | 39 | 84 | 45 |
| 70-74 | 52 | 76 | 24 | 47 | 88 | 41 |
| 75-79 | 49 | 73 | 24 | 52 | 87 | 35 |
| 80-84 | 41 | 66 | 25 | 51 | 83 | 32 |
| 85+ | 33 | 51 | 18 | 43 | 71 | 28 |
| Overall | 39 | 61 | 22 | 46 | 78 | 32 |
| **High intensity statin** | **Dementia** | | | **No Dementia** | | |
| Age group | Before (%) | After (%) | Change (%-point) | Before (%) | After (%) | Change (%-point) |
| 30-64 | 27 | 62 | 35 | 17 | 70 | 53 |
| 65-69 | 23 | 52 | 29 | 22 | 73 | 51 |
| 70-74 | 25 | 54 | 29 | 21 | 70 | 49 |
| 75-79 | 22 | 52 | 30 | 23 | 68 | 45 |
| 80-84 | 16 | 45 | 29 | 23 | 62 | 39 |
| 85+ | 11 | 31 | 20 | 14 | 49 | 35 |
| Overall | 16 | 41 | 25 | 19 | 59 | 40 |
| **Low-Moderate**  **intensity statin** | **Dementia** | | | **No Dementia** | | |
| Age group | Before (%) | After (%) | Change (%-point) | Before (%) | After (%) | Change (%-point) |
| 30-64 | NR* | NR* | NR* | 12 | 11 | -1 |
| 65-69 | 23 | 19 | -4 | 18 | 11 | -7 |
| 70-74 | 27 | 22 | -5 | 26 | 18 | -8 |
| 75-79 | 27 | 22 | -5 | 29 | 19 | -10 |
| 80-84 | 25 | 21 | -4 | 28 | 21 | -8 |
| 85+ | 22 | 19 | -3 | 29 | 22 | -7 |
| Overall | 23 | 20 | -3 | 27 | 20 | -7 |
| **No statin** | **Dementia** | | | **No Dementia** | | |
| Age group | Before (%) | After (%) | Change (%-point) | Before (%) | After (%) | Change (%-point) |
| 30-64 | 67 | 32 | -35 | 71 | 18 | -53 |
| 65-69 | 54 | 29 | -24 | 61 | 16 | -45 |
| 70-74 | 48 | 24 | -24 | 53 | 12 | -41 |
| 75-79 | 51 | 27 | -26 | 48 | 13 | -35 |
| 80-84 | 59 | 34 | -25 | 49 | 17 | -32 |
| 85+ | 67 | 49 | -18 | 57 | 29 | -28 |
| Overall | 61 | 39 | -22 | 54 | 22 | -32 |

*NR=not reported due to low patient numbers in group

### Table S5. Consistency of statin intensity before and after IS

| **Statin intensity before IS** | **Statin intensity after IS, n (%)** | | | | | | | |
| --- | --- | --- | --- | --- | --- | --- | --- | --- |
|  | **Dementia N=2221** | | | | **No Dementia N=8884** | | | |
|  | High | Low-mod | None | Total | High | Low-mod | None | Total |
| High | 298 (82.6) | 14 (3.9) | 49 (13.6) | 361 | 1484 (89.6) | 48 (2.9) | 124 (7.5) | 1656 |
| Low-mod | 134 (26.1) | 315 (61.3) | 65 (12.7) | 514 | 1009 (41.5) | 1209 (49.7) | 214 (8.8) | 2432 |
| None | 488 (36.3) | 110 (8.2) | 748 (55.6) | 1346 | 2706 (56.4) | 514 (29.0) | 1576 (32.9) | 4796 |

IS=ischaemic stroke; low-mod=low-moderate

### Table S6. Ezetimibe as monotherapy after IS

|  | **Dementia N=2221** | **No Dementia N=8884** |
| --- | --- | --- |
| **Statin use after IS = ‘none’, n** | 862 | 1914 |
| **Ezetimibe use in 1st 60 days after IS where Statin use after IS = ‘none’, n (%)** | 12 (1.4%) | 52 (2.7%) |

Ezetimibe use=PBS supply of ezetimibe 10mg tablets; IS=ischaemic stroke

### Table S7. Statin intensity after ischaemic stroke by discharge destination

|  | **Aged Care** | | | | |  |
| --- | --- | --- | --- | --- | --- | --- |
| **Statin intensity,**  **n (%)** | **Dementia**  **(N=717)** | |  | **No dementia**  **(N=888)** | | **Difference**  **(%-point)** |
| High | 247 | (34.5) |  | 384 | (43.2) | -8.7 |
| Low-moderate | 115 | (16.0) |  | 189 | (21.3) | -5.3 |
| None | 355 | (49.5) |  | 315 | (35.5) | 14.0 |
|  | **Private residence** | | | | |  |
|  | **Dementia (N=1317)** | |  | **No dementia**  **(N=7346)** | |  |
| High | 596 | (45.3) |  | 4492 | (61.2) | -15.9 |
| Low-moderate | 284 | (21.8) |  | 1442 | (19.6) | 2.2 |
| None | 434 | (33.0) |  | 1411 | (19.2) | 13.8 |
|  | **Other care setting** | | | | |  |
|  | **Dementia**  **(N=187)** | |  | **No dementia**  **(N=650)** | |  |
| High | 77 | (41.2) |  | 323 | (49.7) | -8.5 |
| Low-moderate | 37 | (19.8) |  | 139 | (21.4) | -1.6 |
| None | 73 | (39.0) |  | 188 | (28.9) | 10.1 |

N=total in group; n=number in subgroup

### Table S8. Odds of using a high intensity statin compared to no statin after ischaemic stroke

| **Predictor** | **Unadjusted model** | |  | **Adjusted model** | |  |
| --- | --- | --- | --- | --- | --- | --- |
|  | **OR** | **95%CI** |  | **OR** | **95%CI** |  |
| Dementia | 0.393 | 0.353-0.437 |  | 0.458 | 0.405-0.518 |  |
| Female vs male | 0.558 | 0.509-0.611 |  | 0.723 | 0.650-0.805 |  |
| Age group |  |  |  |  |  |  |
| 30-64 vs 65-69 | 0.893 | 0.647-1.233 |  | 0.939 | 0.668-1.320 |  |
| 70-74 vs 65-69 | 1.305 | 0.959-1.776 |  | 1.325 | 0.956-1.836 |  |
| 75-79 vs 65-69 | 1.153 | 0.883-1.505 |  | 1.109 | 0.835-1.474 |  |
| 80-84 vs 65-69 | 0.771 | 0.601-0.989 |  | 0.811 | 0.622-1.059 |  |
| 85+ vs 65-69 | 0.375 | 0.296-0.476 |  | 0.460 | 0.356-0.595 |  |
| Charlson Comorbidity Index score | 0.938 | 0.917-0.960 |  | 0.912 | 0.888-0.936 |  |
| Previous stroke^ | 0.810 | 0.688-0.953 |  | 0.628 | 0.520-0.758 |  |
| Married vs single* (32 missing) | 1.684 | 1.538-1.845 |  | 1.137 | 1.021-1.266 |  |
| Regional residence vs metro | 0.805 | 0.729-0.888 |  | 0.773 | 0.691-0.865 |  |
| Public vs private patient | 1.203 | 1.094-1.322 |  | 1.381 | 1.239-1.538 |  |
| Interpreter yes vs no (22 missing) | 1.205 | 1.032-1.407 |  | 1.251 | 1.048-1.494 |  |
| Discharge destination |  |  |  |  |  |  |
| Aged care vs private residence | 0.342 | 0.303-0.385 |  | 0.563 | 0.489-0.648 |  |
| Other care vs private residence | 0.556 | 0.471-0.655 |  | 0.745 | 0.622-0.893 |  |
| Prior statin |  |  |  |  |  |  |
| Prior high intensity vs none | 7.495 | 6.355-8.839 |  | 7.774 | 6.534-9.250 |  |
| Prior low-mod vs none | 2.981 | 2.588-3.434 |  | 3.020 | 2.607-3.499 |  |
| Prior high vs low-mod~ | 2.514 | 2.051-3.082 |  | 2.574 | 2.084-3.179 |  |

OR=Odds ratio; 95%CI=95% Confidence interval;

^previous stroke=record of previous admission for IS in dataset.

*Married=married/de Facto; Single=never married, divorced, separated, or widowed. ~multinomial logistic regression model rerun with difference reference group.

### Table S9. Odds of using a high intensity statin compared to low-moderate intensity statin after ischaemic stroke

| **Predictor** | **Unadjusted model** | |  | **Adjusted model** | |  |
| --- | --- | --- | --- | --- | --- | --- |
|  | **OR** | **95%CI** |  | **OR** | **95%CI** |  |
| Dementia vs no dementia | 0.714 | 0.629-0.810 |  | 0.648 | 0.557-0.754 |  |
| Female vs male | 0.713 | 0.647-0.786 |  | 0.840 | 0.746-0.946 |  |
| Age |  |  |  |  |  |  |
| 30-64 vs 65-69 | 1.242 | 0.825-1.868 |  | 0.923 | 0.587-1.452 |  |
| 70-74 vs 65-69 | 0.638 | 0.461-0.884 |  | 0.689 | 0.475-0.998 |  |
| 75-79 vs 65-69 | 0.605 | 0.450-0.815 |  | 0.689 | 0.490-0.967 |  |
| 80-84 vs 65-69 | 0.509 | 0.382-0.678 |  | 0.567 | 0.408-0.787 |  |
| 85+ vs 65-69 | 0.392 | 0.296-0.518 |  | 0.478 | 0.346-0.659 |  |
| Charlson Comorbidity Index score | 0.955 | 0.932-0.979 |  | 0.958 | 0.930-0.987 |  |
| Previous stroke^ | 0.706 | 0.596-0.836 |  | 0.588 | 0.478-0.724 |  |
| Married vs single (32 missing) | 1.156 | 1.048-1.274 |  | 1.068 | 0.946-1.206 |  |
| Regional Residence vs metro | 0.855 | 0.767-0.952 |  | 0.741 | 0.652-0.841 |  |
| Public vs private patient | 1.395 | 1.261-1.544 |  | 1.457 | 1.292-1.643 |  |
| Interpreter yes vs no (22 missing) | 1.283 | 1.080-1.524 |  | 1.322 | 1.081-1.616 |  |
| Discharge destination |  |  |  |  |  |  |
| Aged care vs private residence | 0.706 | 0.609-0.818 |  | 0.945 | 0.792-1.126 |  |
| Other care vs private residence | 0.773 | 0.642-0.930 |  | 0.950 | 0.768-1.177 |  |
| Prior statin use |  |  |  |  |  |  |
| Prior high vs none | 5.613 | 4.297-7.333 |  | 6.067 | 4.626-7.958 |  |
| Prior low-moderate vs none | 0.147 | 0.131-0.164 |  | 0.150 | 0.133-0.169 |  |
| Prior high vs low-mod~ | 38.31 | 29.40-49.91 |  | 40.420 | 30.92-52.84 |  |

OR=Odds ratio; 95%CI=95% Confidence interval;

^previous stroke=record of previous admission for IS in dataset.

*Married=married/de Facto; Single=never married, divorced, separated, or widowed. ~multinomial logistic regression model rerun with difference reference group.

### Table S10. Odds of using a low-moderate intensity compared to no statin after ischaemic stroke

| **Predictor** | **Unadjusted model** | |  | **Adjusted model** | |  |
| --- | --- | --- | --- | --- | --- | --- |
|  | **OR** | **95%CI** |  | **OR** | **95%CI** |  |
| Dementia | 0.550 | 0.482-0.628 |  | 0.706 | 0.601-0.830 |  |
| Female vs male | 0.782 | 0.698-0.876 |  | 0.861 | 0.750-0.989 |  |
| Age |  |  |  |  |  |  |
| 30-64 vs 65-69 | 0.719 | 0.448-1.155 |  | 1.017 | 0.605-1.709 |  |
| 70-74 vs 65-69 | 2.045 | 2.365-3.066 |  | 1.924 | 1.226-3.021 |  |
| 75-79 vs 65-69 | 1.904 | 1.328-2.731 |  | 1.611 | 1.076-2.413 |  |
| 80-84 vs 65-69 | 1.516 | 1.077-2.134 |  | 1.431 | 0.974-2.102 |  |
| 85+ vs 65-69 | 0.958 | 0.689-1.334 |  | 0.963 | 0.662-1.401 |  |
| Charlson Comorbidity Index score | 0.982 | 0.955-1.011 |  | 0.952 | 0.920-0.985 |  |
| Previous stroke | 1.147 | 0.947-1.389 |  | 1.068 | 0.850-1.342 |  |
| Married vs Single (32 missing) | 1.457 | 1.302-1.631 |  | 1.064 | 0.925-1.225 |  |
| Regional Residence vs metro | 0.942 | 0.834-1.064 |  | 1.044 | 0.903-1.206 |  |
| Public vs private patient | 0.862 | 0.768-0.968 |  | 0.948 | 0.826-1.088 |  |
| Interpreter yes vs no (22 missing) | 0.939 | 0.769-1.148 |  | 0.946 | 0.747-1.200 |  |
| Discharge destination |  |  |  |  |  |  |
| Aged care vs private residence | 0.484 | 0.416-0.563 |  | 0.596 | 0.495-0.717 |  |
| Other care vs private residence | 0.719 | 0.588-0.880 |  | 0.784 | 0.619-0.993 |  |
| Prior statin use |  |  |  |  |  |  |
| Prior high intensity vs none | 1.335 | 0.986-1.808 |  | 1.281 | 0.942-1.743 |  |
| Prior low-moderate vs none | 20.34 | 17.42-23.76 |  | 20.12 | 17.17-23.58 |  |
| Prior high vs low-mod | 0.066 | 0.048-0.090 |  | 0.064 | 0.046-0.088 |  |

OR=Odds ratio; 95%CI=95% Confidence interval;

^previous stroke=record of previous admission for IS in dataset.

*Married=married/de Facto; Single=never married, divorced, separated, or widowed. ~multinomial logistic regression model rerun with difference reference group.

### Table S11. Predictors of statin intensity after ischaemic stroke by dementia status

|  | **With Dementia** | | |  | **Without Dementia** | | |  |
| --- | --- | --- | --- | --- | --- | --- | --- | --- |
| **Predictor** | **High intensity vs none**  **aOR (95% CI)** | **Low-moderate intensity**  **vs none**  **aOR (95% CI)** | **High vs Low-moderate intensity**  **aOR (95% CI)** |  | **High intensity vs none**  **aOR (95% CI)** | **Low-moderate intensity**  **vs none**  **aOR (95% CI)** | **High vs Low-moderate intensity**  **aOR (95% CI)** |  |
| Female vs male | 0.830 (0.666-1.036) | 1.179 (0.869-1.600) | **0.704 (0.526-0.944)** |  | **0.688 (0.609-0.778)** | **0.788 (0.674-0.922)** | **0.873 (0.766-0.995)** |  |
| Age group |  |  |  |  |  |  |  |  |
| 30-64 vs 65-69 | 1.146 (0.577-2.276) | NR | NR |  | 0.897 (0.605-1.330) | 1.252 (0.693-2.261) | 0.716 (0.433-1.186) |  |
| 70-74 vs 65-69 | 1.342 (0.703-2.562) | 1.365 (0.558-3.342) | 0.983 (0.441-2.189) |  | 1.352 (0.923-1.980) | **2.232 (1.312-3.798)** | **0.606 (0.395-0.930)** |  |
| 75-79 vs 65-69 | 1.162 (0.661-2.045) | 1.171 (0.528-2.596) | 0.993 (0.482-2.044) |  | 1.114 (0.800-1.552) | **1.856 (1.151-2.991)** | **0.600 (0.404-0.891)** |  |
| 80-84 vs 65-69 | 0.936 (0.548-1.599) | 1.028 (0.481-2.196) | 0.910 (0.454-1.824) |  | 0.787 (0.578-1.071) | **1.622 (1.030-2.554)** | **0.485 (0.331-0.711)** |  |
| 85+ vs 65-69 | **0.493 (0.293-0.829)** | 0.711 (0.339-1.489) | 0.693 (0.351-1.371) |  | **0.455 (0.339-0.613)** | 1.087 (0.699-1.692) | **0.419 (0.288-0.610)** |  |
| Charlson comorbidity index score | 0.969 (0.919-1.022) | 0.967 (0.900-1.040) | 1.002 (0.934-1.074) |  | **0.894 (0.867-0.922)** | **0.941 (0.906-0.979)** | **0.950 (0.919-0.982)** |  |
| Previous stroke^#^ | 0.739 (0.523-1.044) | 1.352 (0.884-2.068) | **0.547 (0.361-0.827)** |  | **0.584 (0.467-0.731)** | 0.967 (0.735-1.272) | **0.604 (0.474-0.770)** |  |
| Married vs single*^ | 1.194 (0.955-1.492) | 1.320 (0.971-1.796) | 0.904 (0.672-1.217) |  | 1.105 (0.976-1.251) | 0.993 (0.847-1.165) | 1.112 (0.973-1.271) |  |
| Regional residence vs metro | 0.936 (0.732-1.197) | **1.451 (1.044-2.016)** | **0.645 (0.469-0.889)** |  | **0.731 (0.644-0.829)** | 0.954 (0.811-1.121) | **0.766 (0.667-0.881)** |  |
| Public vs private patient | 1.231 (0.972-1.560) | 0.831 (0.608-1.137) | **1.481 (1.093-2.008)** |  | **1.420 (1.257-1.605)** | 0.980 (0.840-1.144) | **1.450 (1.271-1.653)** |  |
| Interpreter yes vs no^ | 0.922 (0.674-1.261) | 0.760 (0.481-1.202) | 1.212 (0.780-1.883) |  | **1.465 (1.172-1.832)** | 1.093 (0.819-1.457) | **1.341 (1.068-1.684)** |  |
| Prior statin intensity |  |  |  |  |  |  |  |  |
| Prior high vs none | **8.434 (6.029-11.798)** | 1.714 (0.907-3.242) | **4.920 (2.739-8.836)** |  | **7.499 (6.124-9.183)** | 1.176 (0.827-1.673) | **6.376 (4.692-8.664)** |  |
| Prior low-mod vs none | **3.062 (2.206-4.248)** | **30.49 (21.65-42.95)** | **0.100 (0.074-0.135)** |  | **2.928 (2.481-3.454)** | **17.81 (14.88-21.32)** | **0.164 (0.114-0.187)** |  |
| Prior high vs low-mod~ | **2.755 (1.785-4.251)** | **0.056 (0.029-0.109)** | **49.00 (27.41-87.59)** |  | **2.562 (2.010-3.264)** | **0.066 (0.046-0.095)** | **38.79 (28.66-52.52)** |  |
| Discharge destination |  |  |  |  |  |  |  |  |
| Aged care vs home | **0.604 (0.480-0.759)** | **0.554 (0.402-0.765)** | 1.089 (0.791-1.500) |  | **0.547 (0.457-0.654)** | **0.624 (0.495-0.786)** | 0.877 (0.708-1.086) |  |
| Other care vs home | 0.861 (0.589-1.261) | 0.864 (0.512-1.457) | 0.997 (0.603-1.651) |  | **0.714 (0.581-0.877)** | **0.759 (0.582-0.991)** | 0.940 (0.742-1.191) |  |

aOR=adjusted odds ratio; CI=Confidence interval; home = private residence; low-mod=low-moderate; NR = not reported due to low patient numbers in group; **Bold** type indicates statistically significant result. ^#^Previous stroke=record of previous admission for ischaemic stroke in dataset. *Married=married/de Facto; Single=never married, divorced, separated, or widowed. ^missing values.

~multinomial logistic regression model rerun with alternative reference group


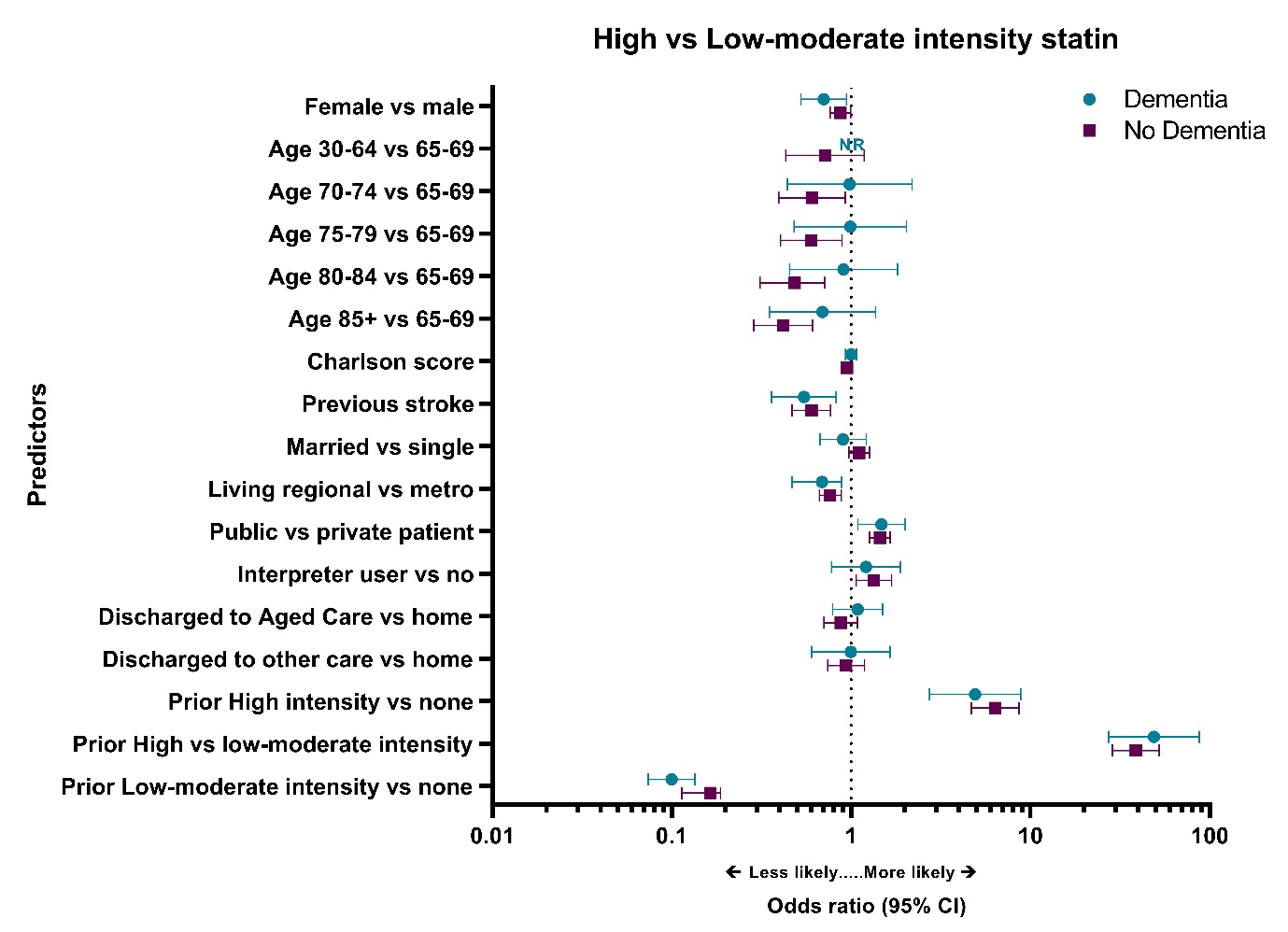


### Figure S1. Predictors of using a high versus a low-moderate intensity statin after ischaemic stroke by dementia status

Charlson score = Charlson Comorbidity Index score; CI = confidence interval; Home = private residence; metro = metropolitan; NR = Not reported for dementia cohort due to low patient numbers in group; See Table S11 for Odds Ratios and 95% CIs.

###
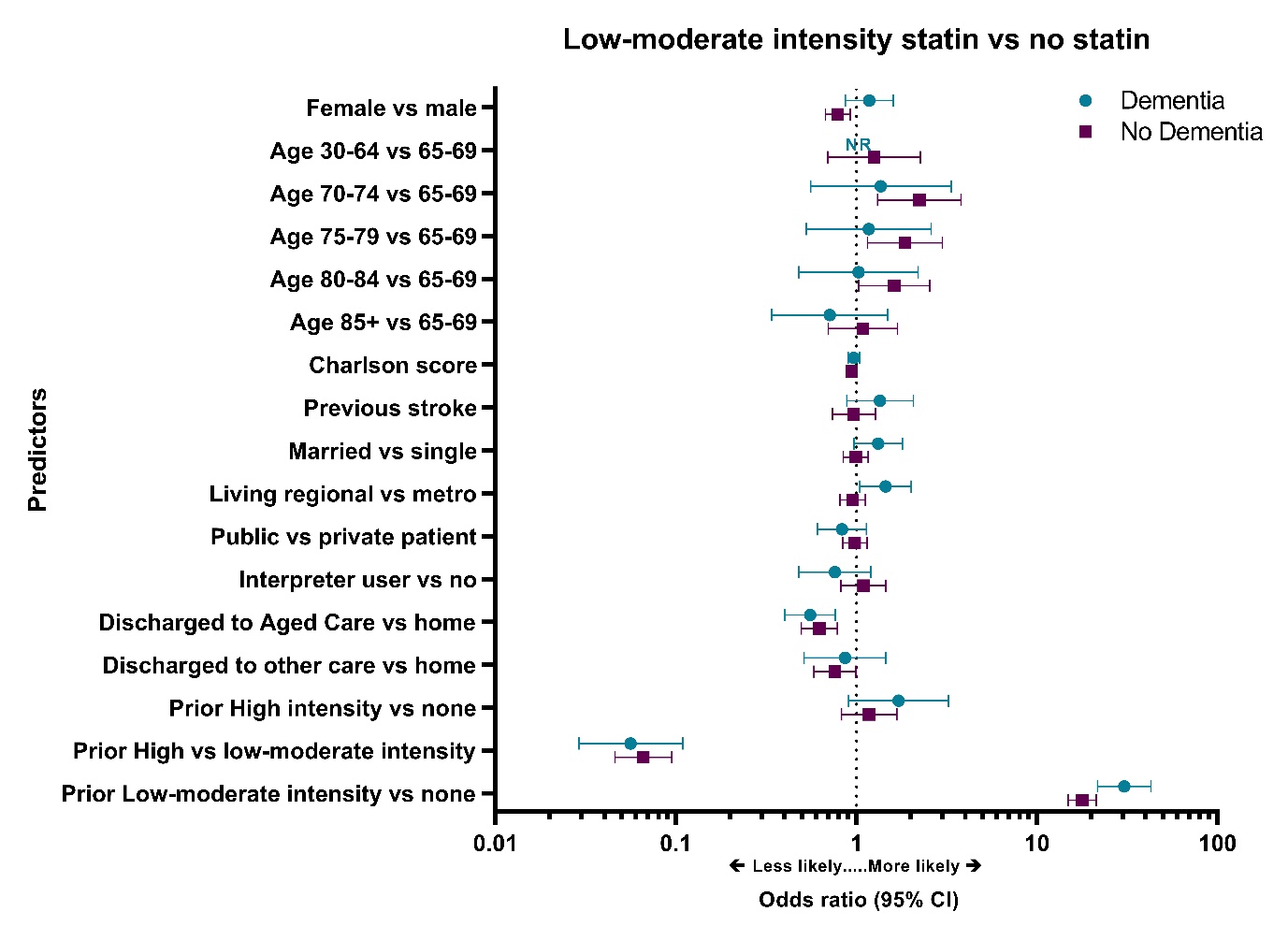
Figure S2. Predictors of using a low-moderate intensity statin vs no statin after ischaemic stroke by dementia status

Charlson score = Charlson Comorbidity Index score; CI = confidence interval; Home = private residence; metro = metropolitan; NR = Not reported for dementia cohort due to low patient numbers in group. See Table S11 for Odds Ratios and 95% CIs.

1. Grundy SM, Stone NJ, Bailey AL, et al. 2018 AHA/ACC/AACVPR/AAPA/ABC/ACPM/ADA/AGS/APhA/ASPC/NLA/PCNA Guideline on the Management of Blood Cholesterol. Journal of the American College of Cardiology 2019;73:e285-e350. doi: doi:10.1016/j.jacc.2018.11.003 [↑](#footnote-ref-1)
